# Indentification of novel *MSTO1* compound heterozygous mutations in a Chinese family with recessive cerebellar atrophy and ataxia

**DOI:** 10.3389/fneur.2022.988519

**Published:** 2022-11-17

**Authors:** Jia Chen, Junfang Xiao, Ge Chen, Qiang Xu, Xingwu Wu, Lifeng Tian, Zhihui Huang, Cailin Xin, Yan Zhao, Zhen Guo, Yang Zou, Qiongfang Wu

**Affiliations:** ^1^Reproductive Medicine Center, Jiangxi Provincial Maternal and Child Health Hospital, Nanchang, China; ^2^Medical Genetics Center, Jiangxi Provincial Maternal and Child Health Hospital, Nanchang, China; ^3^Jiangxi Provincial Key Laboratory of Birth Defect for Prevention and Control, Nanchang, China; ^4^Central Laboratory, Jiangxi Provincial Maxternal and Child Health Hospital, Nanchang, China; ^5^Medical Imaging Center, Jiangxi Provincial Maternal and Child Health Hospital, Nanchang, China

**Keywords:** *MSTO1*, myopathy and ataxia, mitochondrial disorders, whole exome sequencing, missense variant

## Abstract

Misato mitochondrial distribution and morphology regulator 1 (MSTO1) is a nuclear-encoded cytoplasmic protein involved in mitochondrial fusion and distribution. Its disruption causes an extremely rare mitochondrial disorder characterized by early-onset myopathy and cerebellar ataxia. The genotype-phenotype correlation in the *MSTO1* gene is rarely studied before 2017, and only 25 mutations have been described in the patients. Here, we reported two siblings with progressive cerebellar atrophy and ataxia in a Chinese family. Two compound heterozygous mutations in the *MSTO1* gene, a novel missense mutation c.571C>T (p.Arg191Trp), and a reported frameshift mutation c.1259delG (p.Gly420ValfsTer2) were identified in the patients by whole exome sequencing. *in vitro* experiments found both of the mutations lead to reduced protein abundance and link to decreased mtDNA content. Except for ataxia and delayed motor, both of the siblings also have low birth weights, learning difficulties, and dysarthria. Our report enriched the genotype and phenotype spectrums of the *MSTO1*-related disorder and supported the recessive inheritance of the disease.

## Introduction

*MSTO1* is a gene located on chromosome 1q22 [OMIM ^*^617619] and encodes the misato mitochondrial distribution and morphology regulator 1 (MSTO1), which contains a region homologous to the Tubulin/FtsZ GTPase superfamily (1). The specific function of MSTO1 remains unclear. Null mutations of the misato in *Drosophila* are associated with irregular chromosomal segregation at cell division (2–4). The knockdown of MSTO1 by siRNA in HeLa cells resulted in mitochondrial fragmentation and cell death, while overexpression of EGFP-MSTO1 was shown to induce the formation of perinuclear aggregations of mitochondria in COS-7 cells, indicating the critical role of MSTO1 played in mitochondrial distribution and morphology (1).

Mutations in the *MSTO1* gene are associated with a rare disease condition characterized by early-onset myopathy and cerebellar ataxia. To date, only 12 studies (including the current study) have reported 24 families with *MSTO1* mutations. The patients share common clinical symptoms, such as muscle weakness, hypotonia, and ataxia. Rare features including pigmentary retinopathy, arthrogryposis, and scoliosis had also been reported (Table 1) (5–15). Here, we examined a recessively inherited family with two siblings suffering from cerebellar atrophy and ataxia. Two compound heterozygous variants of *MSTO1* (NM_018116.3), c.1259delG (p.G420Vfs^*^2), and c.571C>T (p.R191W) were identified by whole exome sequencing. The c.1259delG variant has been reported in two cases (9, 11), while the c.571C>T variant is described for the first time. According to the 2015 ACMG/AMP classification guidelines (16), the *MSTO1* c.1259delG and c.571C>T variants were classified as “pathogenic” and “likely pathogenic,” respectively. Reduced protein abundance and decreased mtDNA content were observed in both of the variants by *in vitro* experiments. The detailed clinical features of patients with *MSTO1* mutations were reviewed and the genotype–phenotype correlations were discussed. Our study expands the variant diversity of *MSTO1* and supports the recessive inheritance of *MSTO1*-related disease.

**Table 1:** Main clinical and genetic features of the patients with *MSTO1* mutations.

**Cases**	**Patients**	**Affected Familial members**	**Sex/age (years)**	**Symptom onset**	**Mutation**	**Inheritance**	**Plasma CK (U/L)**	**EMG**	**Brain MRI/CT**	**Main symptoms**	**References**
This study	Patient 1	They are siblings.	F/13	2 years	c.571C>T (p.R191W); c.1259delG (p.G420Vfs*2)	AR	1485	Myopathic pattern	Cerebellar hypoplasia, asymmetrical brain ventricles	Ataxia, motor delayed, and learning difficulties	-
	Patient 2		M/11	1 year	c.571C>T (p.R191W); c.1259delG (p.G420Vfs*2)	AR	2194	Myopathic pattern	Atrophy of cerebellum and enlarged cisterna magna	Ataxia, motor delayed, learning difficulties, and slurred speech	
Case 1	Patient 1	She has three affected children.	F/53	38 years	c.22G>A (p.V8M)	AD	Normal range	Myopathic pattern	Frontal atrophy and enlarged interhemispheric fissure	Ataxia, atrophy of muscles, motor and verbal retardation, muscle weakness, pes varus, and short stature (150 cm)	(5)
Case 2	Patient 1	She has an affected sister.	F/17	8–9 months	c.1033C>T (p.R345C); c.1128C>A (p.F376L)	AR	1200	Myopathic pattern	Global cerebellar hypotrophy, enlarged cisterna magna, and hyperintense signals in the supratentorial periventricular and posterior white matter	Myopathy and ataxia, growth and motor retardation, pigmentary retinopathy, scoliosis, asymmetry of the chest, and pectus excavatum	(6)
	Patient 2	-	M/7	1 year	c.971C>T (p.T324I); c.966+1G>A (p.E272_D322 del)	AR	4520	Changes compatible with a myopathic process	Hypoplasia of cerebellar vermis and hemispheres	Myopathy, ataxia, growth and motor retardation, and poor coordination	
Case 3	Patient 1	-	F/13	5 months	c.836G>A (p.R279H); c.1099-1G>A (p.V367Wfs*2)	AR	430	-	Atrophy of cerebellar vermis and hemispheres	Muscle weakness, pigmentary retinopathy, and developmental delay	(7)
	Patient 2	-	F/3	Congenital	c.79C>T (p.Q27*); c.836G>A (p.R279H)	AR	916	-	Atrophy of cerebellar vermis and hemispheres, pons, and tegmental area	Hypotonia, multiple arthrogryposis, and developmental delay	
Case 4	Patient 1	-	M/13	2 years	c.766C>T (p.R256W); c.1435C>T (p.P479S)	AR	909–1614	Normal	Cerebellar atrophy and volume loss	Global developmental delay, proximal weakness, scoliosis, and mild intellectual disability	(8)
Case 5	Patient 1	She has two affected sisters.	F/19	6 months	c.706G>C (p.D236H) ; c.836G>A (p.R279H)	AR	500–788	-	Cerebellar atrophy involving vermis and both hemispheres	Muscle weakness, hypotonia, motor delayed, abnormal gait, speech delay, and short stature	(9)
	Patient 2	He has an affected sister.	M/23	1 year	c.767G>A (p.R256Q); c.1259delG (p.G420Vfs*2)	AR	3487	Myotonia	Cerebellar atrophy involving vermis and both hemispheres	Ataxia, muscle weakness, hypotonia, motor delayed, and dysarthria	
	Patient 3	-	M/37	Congenital	c.651C>G (p.F217L); c.706G>C (p.D236H)	AR	1192	-	-	Muscle weakness, hypotonia, motor delayed, and waddling-like gait	
	Patient 4	-	M/9	Congenital	c.1350G>C (p.L450F); del ex9-14	AR	4387	-	Cerebellar atrophy involving vermis and both hemispheres	Muscle weakness, hypotonia, motor delayed, abnormal gait, and speech delay	
	Patient 5	-	M/6	15 months	c.651C>G (p.F217L); c.706G>C (p.D236H)	AR	600–951	Complex repetitive discharges	Cerebellar atrophy involving vermis and both hemispheres	Muscle weakness, hypotonia, motor delayed, abnormal gait, and dysarthria	
	Patient 6	-	F/19	1 year	c.1433A>G (p.Y478C); Missing second allele	AR?	4029	-	Cerebellar atrophy involving vermis and both hemispheres	Ataxia, muscle weakness, hypotonia, motor delayed, speech delay, esotropia and short stature	
	Patient 7	-	F/16	Congenital	c.707A>G (p.D236G); c.836G>A (p.R279H)	AR	1629	Myotonia	Mild cerebellar atrophy and pontine hypoplasia	Muscle weakness, hypotonia, waddling-like gait, dysarthria, and anisoastigmatism	
	Patient 8	-	F/20	2 years	c.706G>C (p.D236H); c.836G>A (p.R279H)	AR	1200–1300	-	Cerebellar atrophy involving vermis and both hemispheres	Muscle weakness, hypotonia, and abnormal gait	
	Patient 9	-	F/6	1 year	c.971C>T (p.T324I); c.1033C>T (p.R345C)	AR	2249	-	Cerebellar atrophy involving both hemispheres	Ataxia, muscle weakness, hypotonia, motor delayed, dysarthria, short stature, and microcephaly	
	Patient 10	-	M/23	18 months	c.651C>G (p.F217L); c.651C>G (p.F217L)	AR	1450	-	-	Muscle weakness, hypotonia, absent deep tendon reflexes, and wheelchair for longer distances	
	Patient 11	-	M/52	< 7 years	c.651C>G (p.F217L); c.835C>T (p.R279C)	AR	1450	-	-	Muscle weakness, hypotonia, dysarthria, and vasovagal syncope with asystole	
	Patient 12	-	F/7	3 years	c.40G>A (p.G14R); c.225_230del (p.L76S77del)	AR	107	-	Cerebellar atrophy involving vermis and both hemispheres	Ataxia, muscle weakness, hypotonia, dysarthria, and speech delay	
Case 6	Patient 1	-	M/30	Congenital	c.651C>G (p.F217L); del ex7-14	AR	1292	Myopathic pattern	-	Muscle weakness, hypotonia, waddling gait, motor delayed, learning disability, speech difficulty, pectus excavatum, and scoliosis	(10)
Case 7	Patient 1	He has an affected brother.	M/11	1 year	c.836G>A (p.R279H); c.1259delG (p.G420Vfs*2)	AR	2544	Myopathic pattern	Atrophy of cerebellum and brain stem	Muscle weakness, ataxia, psychomotor and mental retardation, pes planus, genu valgus, and short stature (138.4 cm)	(11)
Case 8	Patient 1	-	F/14	1 year	c.22G>A (p.V8M); c.971C>T (p.T324I)	AR	2507	Myopathic pattern, motor neuron involvement	Cerebellar atrophy	Motor delay, frequent falls, slurred speech, hypotonia, muscle weakness, and short stature (150 cm)	(12)
Case 9	Patient1	She has an affected sister.	F/27	2 years	c.1403T>A (p.L468Q); c.1403T>A (p.L468Q)	AR	1000–5200	-	Cerebellar vermian atrophy	Cerebellar ataxia, hypotonia, and dysarthria	(13)
Case 10	Patient1	-	M/1 year and 8 months	9 months	c.13delG (p.A5Pfs*68); c.971C>T (p.T324I)	AR	1544–2803	Myopathic pattern	Cerebellar atrophy	Hypotonia, muscle weakness, and ataxia	(14)
Case 11	Patient1	-	M/3	Infancy	c.1A>G (p.M1?); c.727G>C (p.A243P)	AR	1164	Myopathic pattern	Cerebellar atrophy, deepened sulci and fissures, narrowed gyri, and enlarged fourth ventricle and occipital cisterna	Infancy-onset mental and motor retardation, scoliosis, tremor, bilateral lower extremity muscle weakness, elevated muscle enzymes, and hairy back	(15)

## Patient data

**Figure 1:**
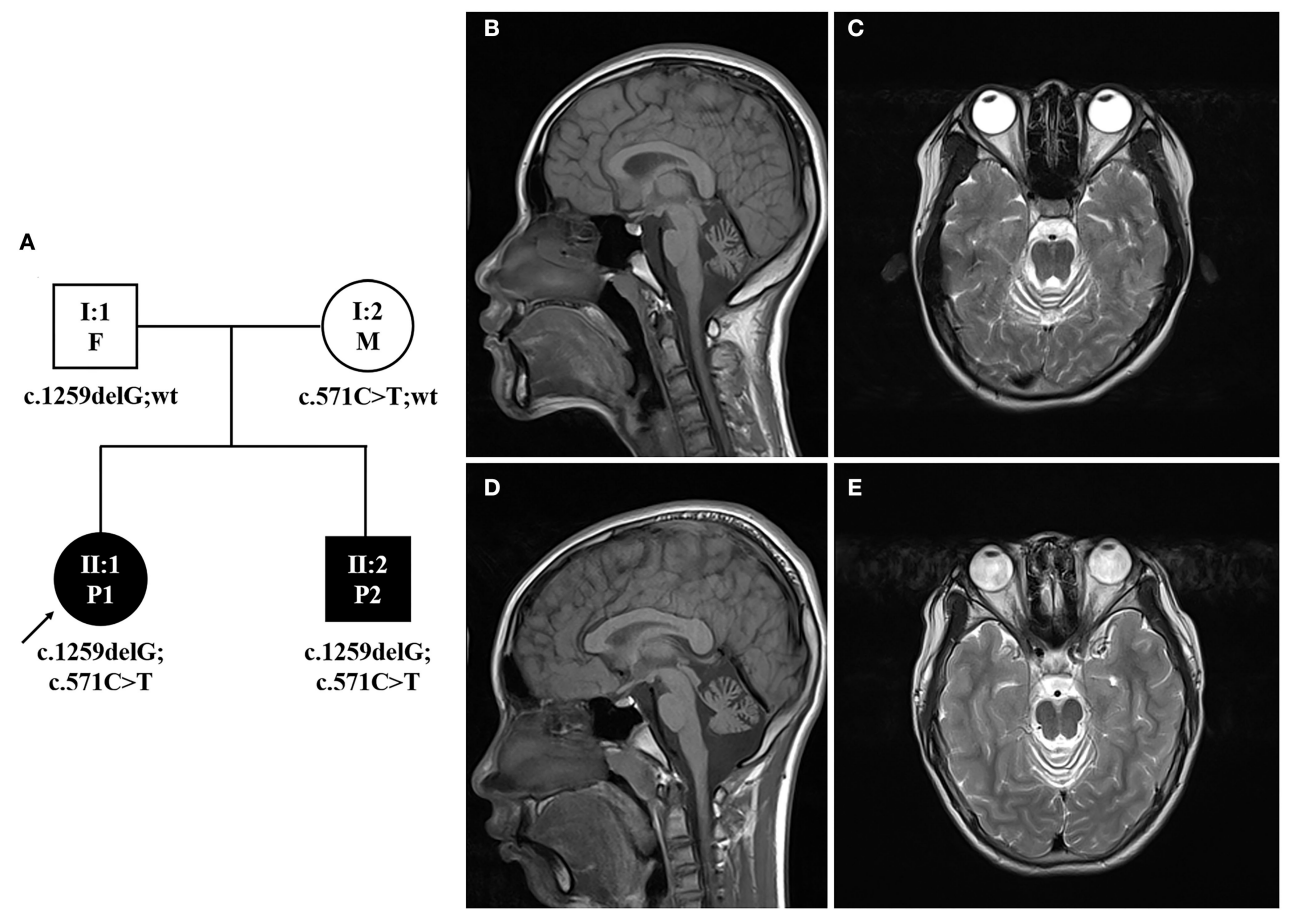
Pedigree and brain MRI of the proband. **(A)** Pedigree of the family with identified *MSTO1* variants. Open symbols, unaffected; filled symbols, affected; squares, male; circles, female; arrow, the proband; F, father; M, mother; P1, patient 1; P2, patient 2. **(B,C)** Brain MRIs of the proband (II:1, P1) showed atrophy of the cerebellum and brain stem, predominantly at the cerebellum. **(D,E)** Brain MRIs of the younger brother of the proband (II:2, P2) showed predominant cerebellar atrophy.

The patients in this study were collected from Jiangxi Provincial Maternal and Child Care Hospital, Nancha, China. Informed consent was obtained from the patient's guardians. A detailed medical history was collected and clinical examinations were performed as part of the standard diagnostic evaluation. The proband (II:1, P1) is the daughter of an unaffected couple. Her younger brother (II:2, P2) was also found to have signs of ataxia. Their parents (I:1, F and I:2, M) declare non-consanguineous and deny family history (Figure 1A).

## Methods

### Ethical approval

All procedures performed in the studies were in accordance with the ethical standards of the Institutional Research Committee. The study was approved by the Medical Ethics Review Board of Jiangxi Maternal and Child Health Hospital, Nanchang, China.

### Whole exome sequencing and analyses

A recessive inheritance pattern was suggested on the basis of unaffected parents giving birth to affected children in the family. To determine the causative mutation in the family, the two affected siblings and their parents were subjected to conduct whole exome sequencing. Genomic DNA was extracted from the peripheral blood lymphocytes using the QIAamp DNA blood mini kit (Qiangen, Germany). The procedures of whole exome sequencing and analysis were performed as previously described (17). The candidate variants of *MSTO1* (NM_018116.3) identified from whole exome sequencing were confirmed by Sanger sequencing.

### Variants interpretation

The pathogenicity of candidate variants was interpreted following the 2015 ACMG/AMP classification guidelines (16). Three software programs were used for *in silico* prediction: MutationTaster (http://www.mutationtaster.org/), PolyPhen-2 (http://genetics.bwh.harvard.edu/pph2/index.shtml), and PROVEAN (http://provean.jcvi.org/seq_submit.php). Only the following outputs were considered as deleterious variants: MutationTaster (disease causing), PolyPhen-2 (probably or possibly damaging), and PROVEAN (deleterious).

### Western blotting and protein abundance detection

The plasmid expressing the wildtype MSTO1 (WT) was constructed by cloning the coding sequence (NM_018116.3) with a HA tag labeled at the C-terminal to the pcDNA3.1(+) vector (Invitrogen, Carlsbad, CA, USA). The plasmids expressing the c.571C>T and the c.1259delG mutants were constructed, respectively, based on the WT plasmid using Mut Express II Fast Mutagenesis Kit V2 (Vazyme, Nanjing, China). One microgram of each plasmid was transfected to 5 × 10^5^ HEK293T cells (CBP60439, Cobioer, Nanjing, China) in 150 mm plates by Lipofectamine 3000 (ThermoFisher, Waltham, MA, USA). After 48 h of transfection, the cells were harvested by trypsin digestion, washed with 1 × phosphate-buffered saline (PBS), and lysed with SDS buffer containing protease inhibitors. Twenty microgram of total cell lysates were resolved on SDS-PAGE gels and transferred onto PVDF membranes, probed with 1:5000 anti-HA (3724, Cell Signaling Technology, Danvers, MA, USA) and 1:5000 anti-GAPDH (10494-1-AP, Proteintech, Rosemont, IL, USA). Blots were visualized by incubating with Clarity ECL substrate (1705061, Bio-Rad, Hercules, CA, USA) and imaging on an Amersham Imager AI600. The gray value of each blot measured by ImageJ software (National Institutes of Health, USA) was used to represent the protein abundance. The relative protein abundance of each genotype was normalized by GAPDH and presented as mean ± SD from at least three independent biological replicates. Unpaired 2-tailed Student's *t*-tests were used to determine statistical significance.

### MtDNA copy number analysis

The plasmids expressing the wildtype, c.571C>T and c.1259delG mutants of MSTO1 were transfected to HEK293T cells, respectively, as described above. The total genomic DNA of the harvested cells was extracted using the QIAamp DNA blood mini kit (Qiangen, Germany). Relative mtDNA copy number was analyzed using the 7500 real-time quantitative PCR System (Applied Biosystems, Foster, CA, USA) and SYBR Green qPCR Master Mix (K0241, Thermo Fisher Scientific, Waltham, MA, USA). Nuclear-encoded housekeeping gene 18S was measured for internal control. The primer sequences specific to mtDNA and 18S were used as described previously (18). At least three independent biological experiments were replicated and statistical significance was determined by unpaired 2-tailed Student's *t*-tests.

## Results

### Clinical data

The clinical features of the two patients are summarized together with previously described patients in Table 1. Patient 1 (II:1) is a 13-year-old girl born after natural birth with a weight of 2.3 kg at 34 weeks. The early onset of symptoms was walking instability with muscle weakness at 2 years old. Furthermore, the patient gradually showed poor motor ability, psycho-motor delay, and low learning ability. Physical examinations revealed normal stature (155 cm) and weight (49 kg) but visual impairment. Muscular atrophy was not obvious. Moderate truncal and lower limb ataxia were present. Serum CK was significantly elevated (1,485 U/L). Blood biochemical examinations revealed the following abnormalities: ALT 55 U/L (< 40), AST 68 U/L (< 40), and LDH 434 U/L (< 245). The brain MRI detected cerebellum hypoplasia, asymmetrical brain ventricles, and small cerebellum tonsils (Figures 1B,C). Electromyography (EMG) examination is considered generalized myopathic damage. No special treatment was given.

Patient 2 (II:2) was the 11 years old younger brother of patient 1. He was born after natural birth with a birth weight of 2.2 kg at 35 weeks. The early onset of walking instability and muscle weakness was also observed at 1 year old. He gradually showed poor fine coordination, slurred speech, and learning difficulties. The physical examination found that his stature was 135 cm and his weight was 34 kg. His visual acuity was within the normal range. Although moderate truncal and lower limb ataxia were also found, his motor ability was better than his affected older sister. Muscular atrophy was observed in the upper limbs (the parents rejected muscle biopsy). Electromyography (EMG) examination revealed myopathic damage. The serum CK was significantly elevated (2,194 U/L). The brain MRI showed a small cerebellum volume, a widening of the cerebellar sulcus, and an expanded cisterna magna (Figures 1D,E).

### Mutation analysis

**Figure 2:**
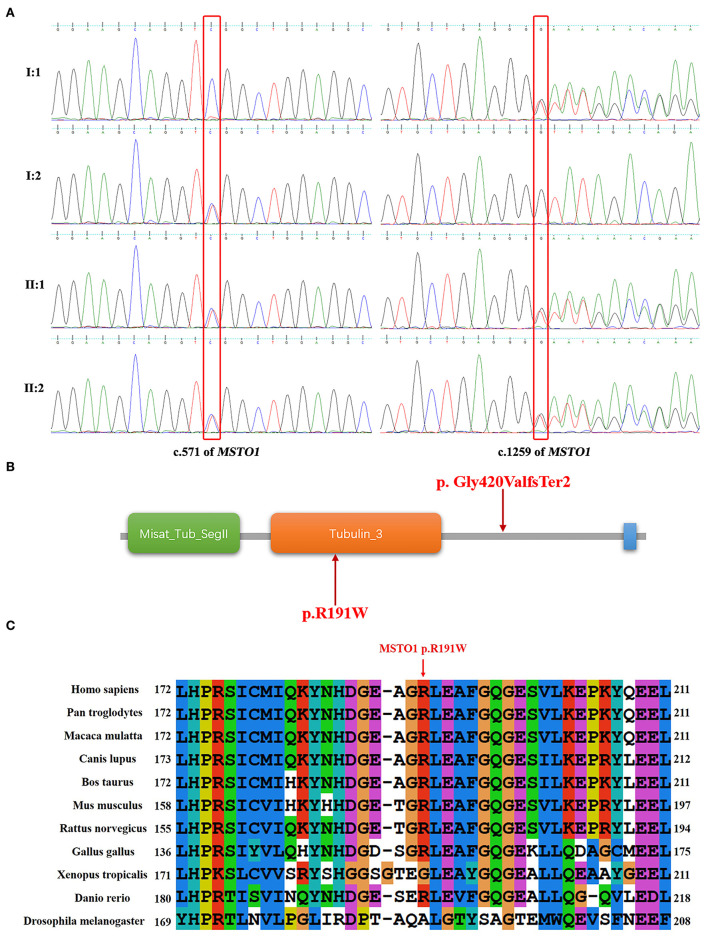
Sequence results of the *MSTO1* variants and analysis of the variants in domains of MSTO1. **(A)** Sequence chromatograms of the c.571C>T and c.1259delG variants of *MSTO1* detected in the family members. **(B)** The domains of the identified MSTO1 variants located. The amino acid change (p.R191W) of the c.571C>T variant is located in the Tubulin domain of the MSTO1 protein. The frameshift variant c.1259delG (p.Gly420ValfsTer2) affected the C-terminal of the protein. **(C)** The affected amino acid R191 of the c.571C>T variant, indicated by a red arrow, is highly conserved in different species.

Whole-exome sequencing analyses of the two patients and their parents were performed to find the genetic cause. Two variants (c.571C>T and c.1259delG) in the *MSTO1* gene (NM_018116.3) and one variant (c.1068dupT) in the *B3GALNT2* gene (NM_152490.2) detected in both of the affected siblings were related to cerebellar atrophy and ataxia. The variant in the *B3GALNT2* gene, also presented in the patient's father, was filtered out because of inconsistency with the phenotypes and inheritance pattern of the family. The c.571C>T (p.Arg191Trp) and c.1259delG (p.Gly420ValfsTer2) variants, inherited from their mother and father, respectively, were considered to contribute to the disease. The variants were verified by the Sanger sequencing in the family members (Figure 2A).

The frameshift variant c.1259delG (p.Gly420ValfsTer2) would generate a premature stop codon located in the 11th of 14 exons of *MSTO1* and encode a truncated protein that may lead to dysfunction of mitochondria. This variant was not observed in the gnomAD (http://gnomad-sg.org/), Exome Sequencing Project (https://evs.gs.washington.edu/EVS/), and 1000 Genomes (https://www.internationalgenome.org/) databases, and had been found in four patients with *MSTO1* related myopathy and ataxia (9, 11). The pathogenicity classification of variants by the 2015 ACMG guidelines indicated that the c.1259delG variant, meeting the criteria PVS1+PS4+PM2, is pathogenic (16).

The other variant c.571C>T (p.Arg191Trp), with the record of rs777651549 in the database of single nucleotide polymorphism (dbSNP, https://www.ncbi.nlm.nih.gov/snp/), was the first time reported in patients with cerebellar ataxia. This variant was not found in the Exome Sequencing Project and 1000 Genomes databases. The allele T had a frequency of 0.00025 (44/174,030) but the absence of homozygotes according to the gnomAD database. The affected amino acid of the c.571C>T variant is located in the Tubulin domain of the MSTO1 protein and is highly conserved in different species (Figures 2B,C). Three online prediction software were used to evaluate the c.571C>T variant and showed consistent results. MutationTaster, PolyPhen-2, and PROVEAN indicated that this variant was “disease causing,” “possibly damaging,” and “deleterious,” respectively. The variant met the criteria PM2+PM3+PP3+PP4 and was classified as “likely pathogenic” (16). We have already submitted the data to ClinVar (https://www.ncbi.nlm.nih.gov/clinvar/). The ClinVar accession for the submission is SCV001190367.

**Figure 3:**
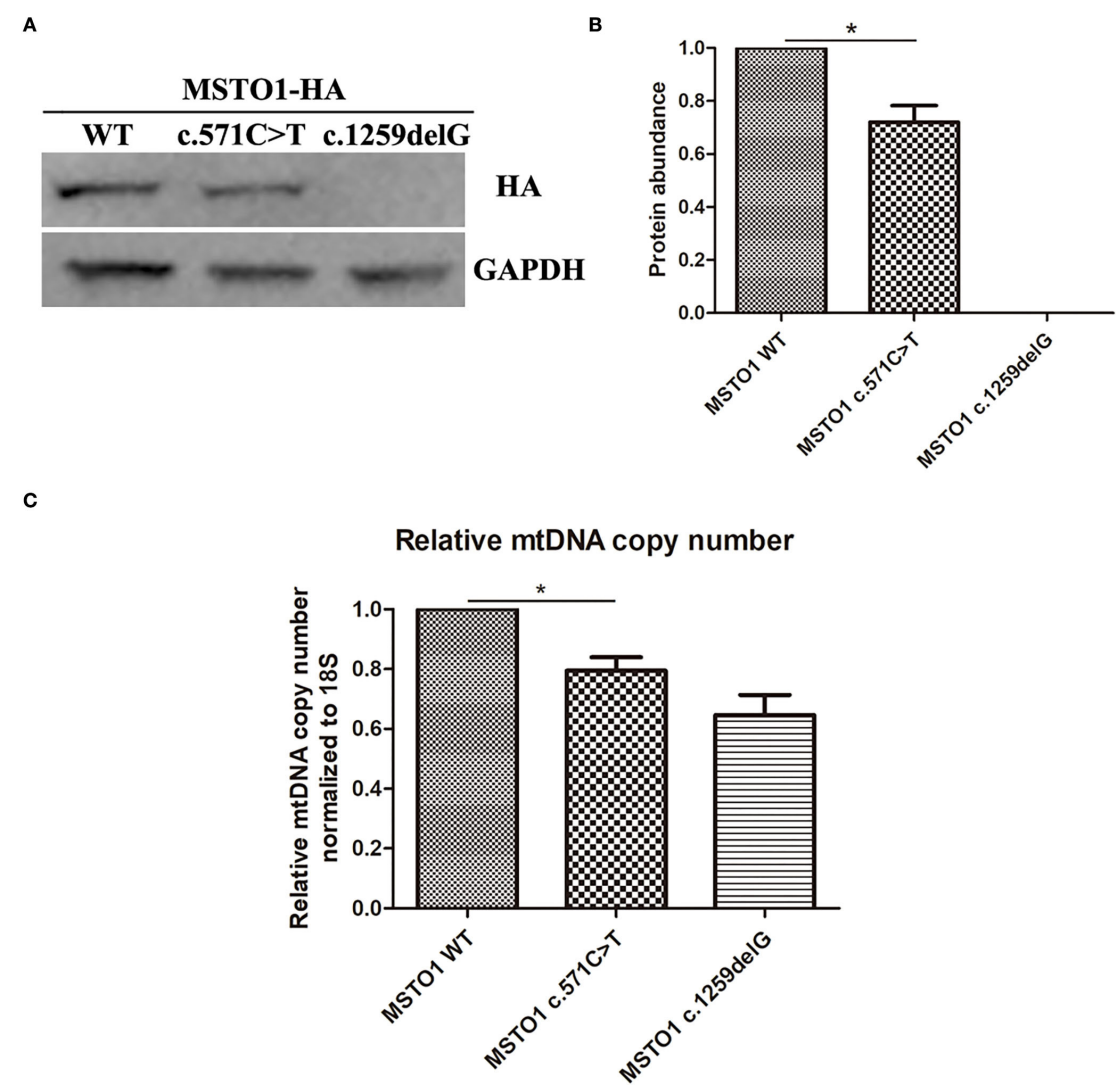
The pathogenic variants in MSTO1 lead to reduced protein abundance and link to decreased mtDNA copy number. **(A)** Western blot analysis of total cell lysates from HEK293T cells overexpressing the HA-labeled wildtype, c.571C>T and c.1259delG mutants of MSTO1 proteins. **(B)** Protein abundance analysis of the wildtype, c.571C>T and c.1259delG mutants of MSTO1 proteins normalized to GAPDH. **(C)** Relative mtDNA copy number normalized to the nuclear-encoded 18S gene. Data represent at least three independent biological replicates. Significance was determined by Student's t test. *P < 0.05.

The effect of the *MSTO1* c.571C>T and c.1259delG variants on protein abundance and mtDNA copy number was evaluated *in vitro*. Plasmids expressing the HA labeled wildtype, c.571C>T, and c.1259delG mutant proteins of MSTO1 were transfected to HEK293T cells, respectively. Western blotting using an anti-HA antibody showed a weakened blot of the c.571C>T mutant and an absent blot of the c.1259delG mutant (Figure 3A). The gray value of each blot measured by the ImageJ software was used to represent the protein abundance. The abundance of the c.571C>T mutant normalized by GAPDH was significantly reduced compared with the wildtype MSTO1 (*P* = 0.047) (Figure 3B). The mtDNA copy number was also detected in those cells. The cells expressing the c.571C>T mutant were found decreased mtDNA copy number compared with the wildtype (*P* = 0.045) (Figure 3C).

## Discussion

*MSTO1*-related ataxia is an extremely rare disease condition among populations. In this study, we presented two patients with novel *MSTO1* compound heterozygous mutations in a Chinese family with cerebellar ataxia. Their phenotypic spectrum overlapped with previously reported patients including cerebellar atrophy, ataxia, myopathy, and learning difficulties. In addition, low birth weights were observed in both patients in our study. Prenatal growth and development conditions of the fetus with *MSTO1* mutations were described by few reports. A Hungarian female patient with the *MSTO1* c.22G>A heterozygous mutation was born as an immature and small baby from an overdue pregnancy (5). Small head circumference at birth was also found in two patients with *MSTO1* mutations (7). These findings indicated that the growth and development delay of patients with *MSTO1* mutations may occur before birth.

MSTO1 is a 570-amino acid cytoplasmic GTPase, containing a Misato segment II tubulin-like domain (6–118 aa) and a tubulin domain (157–346 aa) (13), plays an important role in mitochondrial fusion (5, 6). The novel mutation (p.Arg191Trp) found in our patients lies in the tubulin domain of the MSTO1 protein. Eleven of the 18 reported pathogenic missense mutations (including this case) lie in the tubulin domain (Table 1), suggesting its critical role in pathogenesis. Studies on the fibroblasts of patients with Asp236His/Arg279His, Asp236His/Phe217Leu, and Arg256Gln/Gly420ValfsTer2 mutations revealed that the mutant MSTO1 proteins were undetectable, suggesting mutations in the tubulin domain of MSTO1 affect protein stability (9). In our study, the MSTO1 content of the patients was not tested because the family members did not agree to take a skin biopsy. Our *in vitro* experiments found a significantly reduced protein abundance of the Arg191Trp mutant (Figures 3A,B). It is the first time to reveal a missense mutation on the tubulin domain of MSTO1 has a negative effect on the protein abundance *in vitro*. Our findings supported the important roles of amino acids in the tubulin domain played in the stability of MSTO1.

The mtDNA encodes 13 proteins of the oxidative phosphorylation machinery that are essential for ATP production (19). MtDNA depletion could be a serious consequence caused by disorders of mitochondrial fusion and fission. Fragmented mitochondria and decreased mtDNA content were observed in patients with *MSTO1* mutations, indicating the critical function of MSTO1 in maintaining mitochondrial fusion (9). Our *in vitro* experiments found decreased mtDNA copy number in the cells expressing the Arg191Trp mutant (Figure 3C), demonstrating the mutant impaired the normal function of MSTO1. This impairment could be attributed to reducing the protein abundance and disrupting mitochondrial fusion.

The majority of mutations of *MSTO1* were identified in autosomal recessive inherited families except for the c.22G>A (p.Val8Met) variant causing a dominant inheritance (Table 1). The phenotypic features of patients in dominant and recessive modes were different. Most patients with biallelic *MSTO1* mutations have early symptom onset ages (within 1 or 2 years), elevated plasma CK levels, and atrophied cerebellum. While late onset ages (15–53 years old), normal CK levels and absence of cerebellar atrophy were observed in patients carrying the dominant variant (Table 1). Despite the phenotypic discrepancy that occurs, similar molecular and cellular biological changes, such as decreased protein levels and fragmented mitochondria were discovered in both dominant and recessive patients (5, 6, 9). The mode of inheritance may depend on the location of the variant or the difference in the genetic condition. The variants reported in our case supported the recessive inheritance mode.

In conclusion, we identified novel compound heterozygous mutations in a Chinese family with two siblings suffering from cerebellar atrophy and ataxia. Our study broadens the mutation spectrum of the MSTO1 gene and supports the recessive inheritance pattern of the disease.

## Data availability statement

The datasets presented in this study can be found in online repositories. The name of the repository and accession number can be found below: Figshare, https://figshare.com/, https://doi.org/10.6084/m9.figshare.20362797.v1.

## Ethics statement

The studies involving human participants were reviewed and approved by medical ethics review board of Jiangxi maternal and child health hospital. Written informed consent to participate in this study was provided by the participants' legal guardian/next of kin. Written informed consent was obtained from the individual(s), and minor(s)' legal guardian/next of kin, for the publication of any potentially identifiable images or data included in this article.

## Author contributions

QW, YZo, and JC designed the study. JC and JX drafted the manuscript. GC, QX, and XW performed the DNA extraction, PCR, and sequencing experiments. LT, ZH, CX, YZh, and ZG collected the clinical information. All authors have read and approved the final manuscript.

## Funding

This study was supported by the National Natural Science Foundation of China (Grant 82160317), the Key Research and Development Program of Jiangxi Province (No.20192BBGL70006), and the Application and Cultivation Program of Jiangxi Province (No.20212BAG70007).

## Conflict of interest

The authors declare that the research was conducted in the absence of any commercial or financial relationships that could be construed as a potential conflict of interest.

## Publisher's note

All claims expressed in this article are solely those of the authors and do not necessarily represent those of their affiliated organizations, or those of the publisher, the editors and the reviewers. Any product that may be evaluated in this article, or claim that may be made by its manufacturer, is not guaranteed or endorsed by the publisher.

## References

[1] Kimura M Okano Y. Human Misato regulates mitochondrial distribution and morphology. Exp Cell Res. (2007) 313:1393–404. 10.1016/j.yexcr.2007.02.004 17349998

[2] Miklos GL Yamamoto M Burns RG Maleszka R. An essential cell division gene of Drosophila, absent from Saccharomyces, encodes an unusual protein with tubulin-like and myosin-like peptide motifs. Proc Natl Acad Sci U S A. (1997) 94:5189–94. 10.1073/pnas.94.10.5189 9144213 PMC24654

[3] Gurvitz A Hartig A Ruis H Hamilton B de Couet HG. Preliminary characterisation of DML1, an essential Saccharomyces cerevisiae gene related to misato of Drosophila melanogaster. FEMS Yeast Res. (2002) 2:123–35. 10.1111/j.1567-1364.2002.tb00077.x 12702300

[4] Mottier-Pavie V Cenci G Vernì F Gatti M Bonaccorsi S. Phenotypic analysis of misato function reveals roles of noncentrosomal microtubules in Drosophila spindle formation. J Cell Sci. (2011) 124 (Pt 5):706–17. 10.1242/jcs.072348 21285248

[5] Gal A Balicza P Weaver D Naghdi S Joseph SK Várnai P. MSTO1 is a cytoplasmic pro-mitochondrial fusion protein, whose mutation induces myopathy and ataxia in humans. EMBO Mol Med. (2017) 9:967–84. 10.15252/emmm.201607058 28554942 PMC5494519

[6] Nasca A Scotton C Zaharieva I Neri M Selvatici R Magnusson OT. Recessive mutations in MSTO1 cause mitochondrial dynamics impairment, leading to myopathy and ataxia. Hum Mutat. (2017) 38:970–7. 10.1002/humu.23262 28544275 PMC5575512

[7] Iwama K Takaori T Fukushima A Tohyama J Ishiyama A Ohba C. Novel recessive mutations in MSTO1 cause cerebellar atrophy with pigmentary retinopathy. J Hum Genet. (2018) 63:263–70. 10.1038/s10038-017-0405-8 29339779

[8] Ardicli D Sarkozy A Zaharieva I Deshpande C Bodi I Siddiqui A. A novel case of MSTO1 gene related congenital muscular dystrophy with progressive neurological involvement. Neuromuscul Disord. (2019) 29:448–55. 10.1016/j.nmd.2019.03.011 31130378

[9] Donkervoort S Sabouny R Yun P Gauquelin L Chao KR Hu Y. MSTO1 mutations cause mtDNA depletion, manifesting as muscular dystrophy with cerebellar involvement. Acta Neuropathol. (2019) 138:1013–31. 10.1007/s00401-019-02059-z 31463572 PMC6851037

[10] Schultz-Rogers L Ferrer A Dsouza NR Zimmermann MT Smith BE Klee EW. Novel biallelic variants in MSTO1 associated with mitochondrial myopathy. Cold Spring Harb Mol Case Stud. (2019) 5:a004309. 10.1101/mcs.a004309 31604776 PMC6913144

[11] Li K Jin R Wu X. Whole-exome sequencing identifies rare compound heterozygous mutations in the MSTO1 gene associated with cerebellar ataxia and myopathy. Eur J Med Genet. (2020) 63:103623. 10.1016/j.ejmg.2019.01.013 30684668

[12] Jiao Y Kuang S Yang S Han X. Evidence of motor axon or motor neuron damage in a Chinese patient with compound heterozygous MSTO1 variants. Acta Neurol Belg. (2021) 121:795–97. 10.1007/s13760-020-01544-7 33222031

[13] Nasca A Di Meo I Fellig Y Saada A Elpeleg O Ghezzi D. A novel homozygous MSTO1 mutation in Ashkenazi Jewish siblings with ataxia and myopathy. J Hum Genet. (2021) 66:835–40. 10.1038/s10038-020-00897-4 33612823

[14] Tian Y Shi Z Hou C Li W Zhu H Li X. Diagnosis of a child with mitochondrial myopathy and cerebellar atrophy with ataxia due to compound heterozygous variants of MSTO1 gene. Zhonghua Yi Xue Yi Chuan Xue Za Zhi. (2022) 39:417–20. 10.3760/cma.j.cn511374-20210319-00249 35446979

[15] Liu L Su R Huang P Li X Xiong J Xiao Y. Case Report: Evidences of myasthenia and cerebellar atrophy in a chinese patient with novel compound heterozygous MSTO1 variants. Front Genet. (2022) 13:947886. 10.3389/fgene.2022.947886 36035138 PMC9402982

[16] Richards S Aziz N Bale S Bick D Das S Gastier-Foster J. Standards and guidelines for the interpretation of sequence variants: a joint consensus recommendation of the American College of Medical Genetics and Genomics and the Association for Molecular Pathology. Genet Med. (2015) 17:405–24. 10.1038/gim.2015.30 25741868 PMC4544753

[17] Chen J Yuan H Xie K Wang X Tan L Zou Y. A novel TAB2 nonsense mutation (pS149X) causing autosomal dominant congenital heart defects: a case report of a Chinese family. BMC Cardiovasc Disord. (2020) 20:27. 10.1186/s12872-019-01322-1 31959127 PMC6971906

[18] Eaton JS Lin ZP Sartorelli AC Bonawitz ND Shadel GS. Ataxia-telangiectasia mutated kinase regulates ribonucleotide reductase and mitochondrial homeostasis. J Clin Invest. (2007) 117:2723–34. 10.1172/JCI31604 17786248 PMC1952633

[19] Nunnari J Suomalainen A. Mitochondria: in sickness and in health. Cell. (2012) 148:1145–59. 10.1016/j.cell.2012.02.035 22424226 PMC5381524

